# Left Atrial to Left Ventricular Bypass Using a Valved Conduit for Mitral Prosthesis–Patient Mismatch

**DOI:** 10.1016/j.atssr.2023.06.002

**Published:** 2023-06-26

**Authors:** Brett F. Curran, Hartzell V. Schaff

**Affiliations:** 1 Department of Cardiovascular Surgery, Mayo Clinic, Rochester, Minnesota

## Abstract

A 72-year-old woman presented with dyspnea 2 years after mitral valve replacement with a 25-mm Epic bioprosthesis. Exercise echocardiography revealed a mean transvalvular gradient of 16 mm Hg, consistent with functional mitral stenosis due to prosthesis-patient mismatch. Because of the anticipated difficulties with insertion of a larger prosthesis, we proceeded with bypass of the mitral valve using a left atrial to left ventricular valved conduit. The patient had resolution of symptoms, and imaging demonstrated a widely patent graft. Left atrial to left ventricular bypass using a valved conduit is an effective treatment of mitral prosthesis–patient mismatch in the setting of severe mitral annular calcification.

Mitral prosthesis–patient mismatch (PPM) is assumed to be present when the transvalvular gradient is increased in prostheses with normal leaflet function. PPM has been defined as an indexed effective orifice area (EOAi) of ≤1.2 cm^2^/m^2^.^1^ The resulting functional mitral stenosis leads to increased left atrial and pulmonary arterial pressures and, in some patients, right-sided heart failure.^1^^,^^2^ More commonly, these patients present with exertional symptoms and are predisposed to atrial fibrillation.^1^

Valve re-replacement with a larger or more hemodynamically efficient prosthesis is the preferred approach for most patients. However, mitral valve replacement for mitral PPM may be difficult or hazardous because of poor access and exposure of the valve or severe mitral annular calcification. This report describes mitral valve bypass with a left atrial to left ventricular valved conduit for management of mitral PPM.

The patient is a 72-year-old woman who underwent mitral valve replacement at an outside institution in 2017 for mitral stenosis. The operating surgeon described severe mitral annular calcification with a large bar of calcium involving the annulus circumferentially as well as the leaflets. After débridement of the annular calcium, a 25-mm St Jude Epic bioprosthesis (Abbott Medical) was implanted. The manufacturer’s reported mean effective orifice area for the prosthesis is 1.2 cm^2^, and the EOAi for the patient using her body surface area of 1.69 m^2^ was 0.71 cm^2^/m^2^.

Initially after the operation she was able to ambulate up to a mile without difficulty, but by the time she presented to our institution in 2019, she was able to walk only 1 block before dyspnea developed. Transesophageal echocardiography revealed a mitral inflow gradient of 10 mm Hg with normal appearance and motion of bioprosthetic valve leaflets. Computed tomography (CT) of the chest showed dense mitral annular calcification around the mitral prosthesis (Figure 1). The patient underwent a stress test that demonstrated a mean mitral prosthetic valve gradient of 6 mm Hg at rest that increased to 16 mm Hg at peak stress (Figure 2). With exercise, she also had an increase in pulmonary artery pressures to 64 mm Hg with the development of lung interstitial edema. Cardiac catheterization showed no important coronary artery disease. The patient’s exercise-induced elevated mitral gradient with corresponding increases in pulmonary artery pressure in the setting of normal prosthetic valve cusp pliability was consistent with mitral PPM.

**Figure 1:**
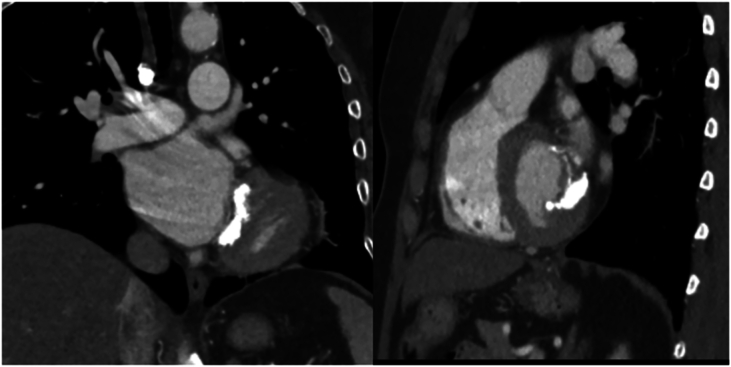
Computed tomography images of the chest demonstrating bioprosthetic mitral valve with dense mitral annular calcification.

**Figure 2:**
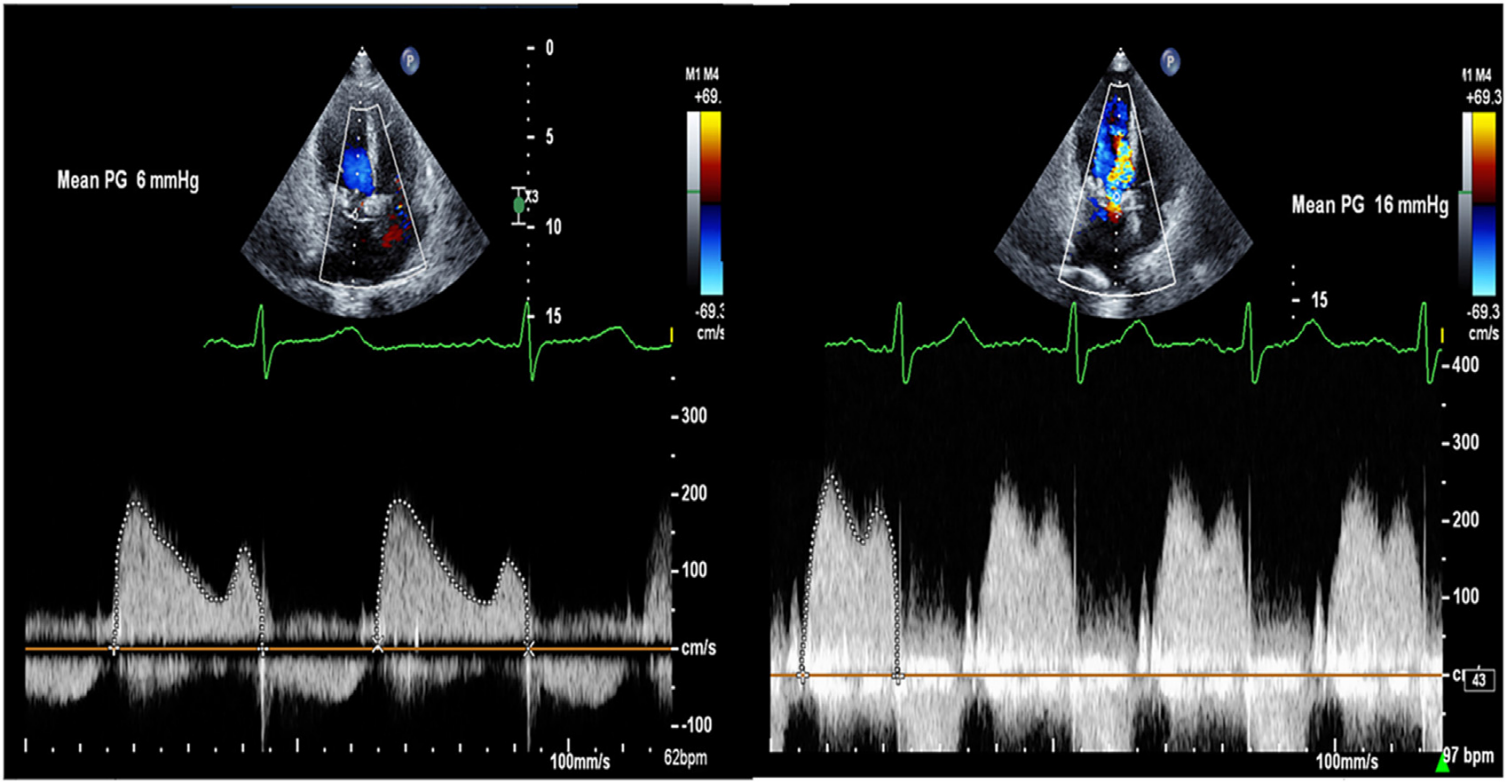
Preoperative stress test echocardiogram demonstrating mean mitral valve gradient of 6 mm Hg at rest and 16 mm Hg at peak stress. (PG, prosthetic valve gradient.)

The operation was performed through a secondary median sternotomy with standard cannulation for normothermic cardiopulmonary bypass using aortic and right atrial cannulas. After cardioplegic cardiac arrest (total of 52 minutes), the mitral valve was inspected through a standard left atriotomy posterior to the interatrial groove. The prosthetic valve leaflets were pliable and had normal excursions. The valve appeared small for the patient, and there was severe annular calcification. Because of this calcification, excision of the prosthesis and annular enlargement for insertion of a larger valve seemed hazardous. Therefore, the left atriotomy was closed and we proceeded with a bypass of the mitral valve with a left atrial to left ventricular valved conduit.

The left atrial appendage was exposed and then amputated. The orifice was extended cephalad onto the left superior pulmonary vein. A 26-mm Hemashield graft (Getinge) was beveled and sewn to the orifice of the left atrial appendage with continuous 4-0 Prolene. A 6-cm ventriculotomy was made in the distal lateral wall of the left ventricle near the apex, and a small portion of myocardium was excised to augment the orifice. A 25-mm Medtronic Hancock II bioprosthesis was inserted into the distal end of the conduit and secured with continuous 4-0 Prolene. The distal end of the conduit was beveled and sewn to the ventriculotomy using interrupted 2-0 Prolene sutures backed with felt pledgets. The initial layer of interrupted sutures was reinforced with over-and-over 2-0 Prolene.

The patient was easily separated from cardiopulmonary bypass, and transesophageal echocardiography demonstrated laminar flow in the conduit with normal valve function. The left pleural space was opened widely, and the pericardium was incised to prevent any compression of the conduit.

Postoperative recovery was uncomplicated, and transthoracic Doppler echocardiography performed on postoperative day 5 confirmed a widely patent conduit with a mean gradient of 2 mm Hg. CT angiography on the sixth postoperative day showed normal flow and configuration of the valved conduit. Similar findings were present on repeated chest CT angiography performed 3 months after the operation (Figure 3). Eighteen months postoperatively, she is active with no cardiac symptoms.

**Figure 3:**
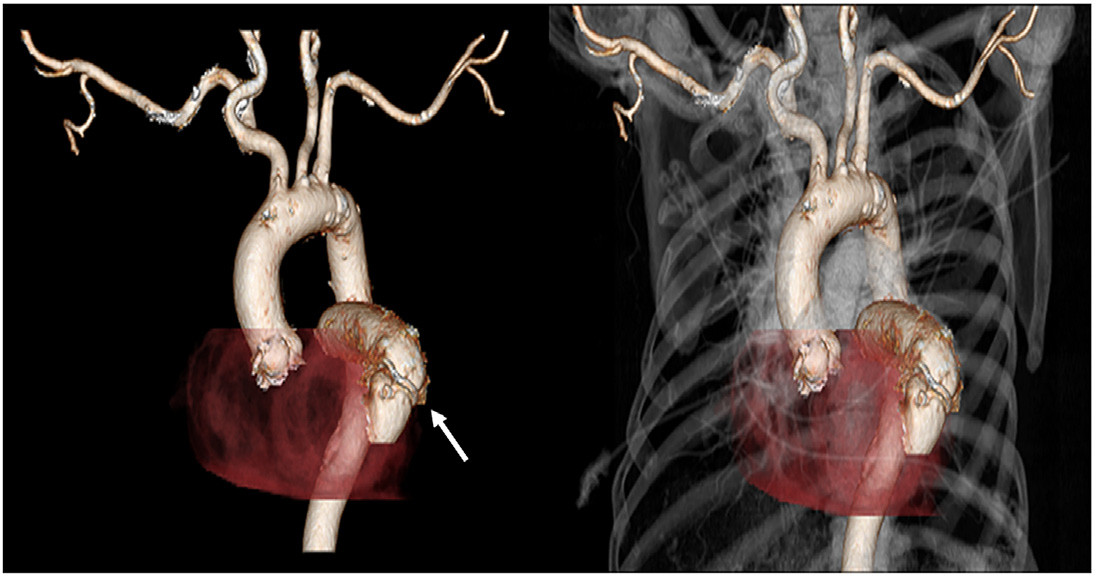
Postoperative computed tomography angiogram of the chest demonstrates a widely patent left atrial to left ventricular bypass. Note the proximity of the prosthetic valve to the ventricular anastomosis (arrow).

## Comment

The reported incidence of mitral PPM after mitral valve replacement is variable. Magne and coworkers^1^ estimated that the incidence of mitral PPM, EOAi of ≤1.2 cm^2^/m^2^, is approximately 78%. PPM is a strong independent risk factor for the persistence of pulmonary hypertension and congestive heart failure after mitral valve replacement.^2^ In some reports, patients with mitral valve PPM, especially those with severe PPM, EOAi of ≤0.9 cm^2^/m^2^, have reduced late survival compared with those without PPM after mitral valve replacement.^1^^,^^3^ To prevent PPM in the mitral position, it is important to select a prosthesis of adequate size based on the type of valve and the patient’s body surface area. If significant annular calcification prevents implantation of an adequately sized valve, extensive débridement of the annulus can be performed to place a larger prosthesis. Extensive annular débridement, however, can lead to disruption of the atrioventricular groove or injury to the circumflex coronary artery. Procedures to enlarge the mitral annulus are complex. The Manouguian procedure permits enlargement of the mitral and aortic annuli during combined aortic and mitral valve replacement; however, few surgical options are available for isolated mitral valve replacement.^4^

When mitral PPM does occur, treatment options are limited. One strategy is to remove the implanted valve and then aggressively débride any annular calcification with the hope of implanting a larger prosthesis. Marin-Cuartas and coworkers^5^ described a case of mitral PPM due to a small valve implanted in a heavily calcified annulus, similar to the patient in this report. During repeated mitral valve replacement with aggressive mitral annular débridement and reconstruction, a coronary calcification embolism led to the death of the patient. This report highlights the complexity of PPM in the setting of mitral annular calcification.

A valved conduit from the left atrium to the left ventricle to bypass the mitral valve has been employed in the management of severe mitral stenosis secondary to mitral annular calcification.^6^ In this report, we describe the use of a valved conduit from the left atrium to the left ventricle to bypass the mitral valve in the setting of severe mitral valve PPM. This report suggests that left atrial to left ventricular valved conduit bypass is a safe and effective surgical approach to this complex problem for which treatment options are limited and often carry significant risk.
